# Assessment of Retinal Microcirculation in Primary Open-Angle Glaucoma Using Adaptive Optics and OCT Angiography: Correlation with Structural and Functional Damage

**DOI:** 10.3390/jcm14144978

**Published:** 2025-07-14

**Authors:** Anna Zaleska-Żmijewska, Alina Szewczuk, Zbigniew M. Wawrzyniak, Maria Żmijewska, Jacek P. Szaflik

**Affiliations:** 1 Department of Ophthalmology, Public Ophthalmic Clinical Hospital (SPKSO), Medical University of Warsaw, 02-015 Warsaw, Poland; azaleska@wum.edu.pl (A.Z.-Ż.); jacek.szaflik@wum.edu.pl (J.P.S.); 2 Public Ophthalmic Clinical Hospital (SPKSO), 03-709 Warsaw, Poland; alinaszewczuk16@gmail.com; 3 Faculty of Electronics and Information Technology, Warsaw University of Technology, 00-665 Warsaw, Poland; 4 Faculty of Medicine, Student Scientific Society “Eye”, Medical University of Warsaw, 02-097 Warsaw, Poland; maria.zmijewska@student.wum.edu.pl

**Keywords:** primary open-angle glaucoma, retinal microcirculation, adaptive optics, optical coherence tomography angiography, retinal arteriole morphology, vessel density, foveal avascular zone, retinal nerve fibre layer, ganglion cell complex

## Abstract

**Background:** This study aimed to evaluate retinal arteriole parameters using adaptive optics (AO) rtx1™ (Imagine Eyes, Orsay, France) and peripapillary and macular vessel densities with optical coherence tomography angiography (OCTA) in eyes with different stages of primary open-angle glaucoma (POAG) compared to healthy eyes. It also investigated the associations between vascular parameters and glaucoma severity, as defined by structural (OCT) and functional (visual field) changes. **Methods:** Fifty-seven eyes from 31 POAG patients and fifty from 25 healthy volunteers were examined. Retinal arteriole morphology was assessed using the AO rtx1™-fundus camera, which measured lumen diameter, wall thickness, total diameter, wall-to-lumen ratio (WLR), and wall cross-sectional area. OCTA was used to measure vessel densities in superficial (SCP) and deep (DCP) capillary plexuses of the macula and radial peripapillary capillary plexus (RPCP) and FAZ area. Structural OCT parameters (RNFL, GCC, rim area) and visual field tests (MD, PSD) were also performed. **Results:** Glaucoma eyes showed significantly thicker arteriole walls (12.8 ± 1.4 vs. 12.2 ± 1.3 µm; *p* = 0.030), narrower lumens (85.5 ± 10.4 vs. 100.6 ± 11.1 µm; *p* < 0.001), smaller total diameters (111.0 ± 10.4 vs. 124.1 ± 12.4 µm; *p* < 0.001), and higher WLRs (0.301 ± 0.04 vs. 0.238 ± 0.002; *p* < 0.001) than healthy eyes. In glaucoma patients, OCTA revealed significantly reduced vessel densities in SCP (36.39 ± 3.60 vs. 38.46 ± 1.41; *p* < 0.001), DCP (36.39 ± 3.60 vs. 38.46 ± 1.41; *p* < 0.001), and RPCP plexuses (35.42 ± 4.97 vs. 39.27 ± 1.48; *p* < 0.001). The FAZ area was enlarged in eyes with glaucoma (0.546 ± 0.299 vs. 0.295 ± 0.125 mm^2^); *p* < 0.001). Positive correlations were found between vessel densities and OCT parameters (RNFL, *r* = 0.621; GCC, *r* = 0.536; rim area, *r* = 0.489), while negative correlations were observed with visual field deficits (*r* = −0.517). **Conclusions:** Vascular deterioration, assessed by AO rtx1™ and OCTA, correlates closely with structural and functional damage in glaucoma. Retinal microcirculation changes may precede structural abnormalities in the optic nerve head. Both imaging methods enable the earlier detection, staging, and monitoring of glaucoma compared to conventional tests.

## 1. Introduction

Vision deterioration due to glaucoma is a worldwide growing issue. It is estimated that 3% of people over 40 years of age suffer from glaucoma, and this percentage increases several times with age [1]. The extension of life expectancy means that a larger number of glaucoma patients may experience visual field disorders and the resulting dysfunctions in everyday activities. This condition, characterised by retinal ganglion cell (RGC) degeneration and subsequent optic nerve damage, leads to irreversible loss of the visual field (VF) [2,3]. While traditional treatments focus on lowering intraocular pressure (IOP), glaucomatous damage can progress despite IOP reduction, highlighting the importance of understanding the vascular theory of glaucomatous optic neuropathy (GON) [4]. This theory, proposed by Flammer, suggests that impaired ocular blood flow and retinal microvascular autoregulation contribute to the development and progression of the disease [5]. Impaired RGCs require reduced blood supply, causing retinal arteriolar constriction, which has been found even in pre-perimetric glaucoma [6]. The eye’s microvasculature is unique and can be non-invasively and directly visualised, imaged, and quantified. The integrity and function of the retinal tissue depend on adequate perfusion in the microcirculatory network [7,8].

Fluctuation of blood flow and poor ocular perfusion in glaucoma patients correlate with visual field deterioration [7,8]. Glaucoma diagnosis is based on a combination of correlated glaucomatous optic nerve head and visual field defects analyses, followed by monitoring their progression. VF tests are subjective and cannot detect early signs of GON progression. Optical coherence tomography (OCT) examination, including assessment of the peripapillary retinal nerve fibre layer (RNFL) thickness and the ganglion cell complex (GCC), is the gold standard for evaluating structural changes in the optic nerve head (ONH) due to glaucoma (GON) earlier than in perimetric tests. Both perimetry and OCT are crucial but may not detect the earliest stages of glaucoma [9,10].

Newer imaging techniques offer more objective, non-invasive, and detailed insights into retinal microcirculation. The comparison table illustrates the application of emerging imaging techniques for retinal microcirculation analysis, which may be applied in glaucoma diagnosis and monitoring (Table 1).

Scanning laser Doppler flowmetry (SLDF) or dynamic retinal vessel analysis of retinal vascular diameter in response to flickering light allows the examination of dynamic aspects of retinal microcirculation [11,12,13,14]. SLDF enables the investigation of capillary area and retinal capillary flow based on the analysis of perfusion images, as well as the determination of vascular wall thickness, lumen diameter, and the wall-to-lumen ratio (WLR) parameter [11,12,13].

Another new method of measuring the size of small arterioles is adaptive optics (AO). AO enables non-invasive retinal examination at the cellular level with a resolution of approximately two micrometres by measuring wavefront distortions and compensating them in real time with deformable mirrors [13,15,16]. AO technology can be incorporated into imaging devices, such as fundus cameras, optical coherence tomography (OCT), or scanning laser ophthalmoscopy (SLO) [15,16]. Rtx1™ is a commercially available AO-fundus camera (AO-FC) that uses infrared light (850 nm wavelength) and is characterised by a resolution of 1.6 μm. The image dimensions are 4 × 4, representing 1.2 × 1.2 mm of the retina. Image acquisition lasts approximately 4 s, during which 40 individual images are acquired [15]. SLDF and AO can estimate the wall and lumen thickness, as well as WLR and WCSA of retinal arterioles [13]. The reliability of SLDF and AO measurement has been previously confirmed by De Ciuceis C et al. [13].

Development in OCT systems has enabled the non-invasive measurement of retinal vasculature at the optic disc head, peripapillary area, and macular region by determining the dynamic motion of red blood cells in a flowing blood vessel [18,19,20]. This diagnostic technique, introduced in 2016, is known as optical coherence tomography angiography (OCTA) [18]. OCTA images are made by repeating B-scans over the same tissue location, two or more times. Changes in the OCT signal in subsequent scans, caused by the movement of red blood cells or other particles, generate the angiographic contrast with the stable signal from the vessel walls. OCTA devices create a static map of the vascular capillary networks [20]. OCTA can stratify the circulation into inner and deep capillary plexuses [18,19,20]. The superficial network comprises vessels that surround and nourish the nerve fibre and ganglion cell layers. The thickness of this capillary network is significantly greater near the ONH than in other regions of the retina, owing to the increased thickness of the RNFL [20,21]. Peripapillary capillaries arise from arterioles around the optic disc, have few anastomoses, and are very sensitive to IOP fluctuations [20].

Reduced perfusion in the optic nerve head, peripapillary retina, and even the macula has been detected in glaucoma patients using various devices. OCTA has been used to analyse the area of peripapillary microcirculation in glaucoma patients and has demonstrated strong diagnostic accuracy, repeatability, and reproducibility [21,22,23,24,25].

**Figure 1:**
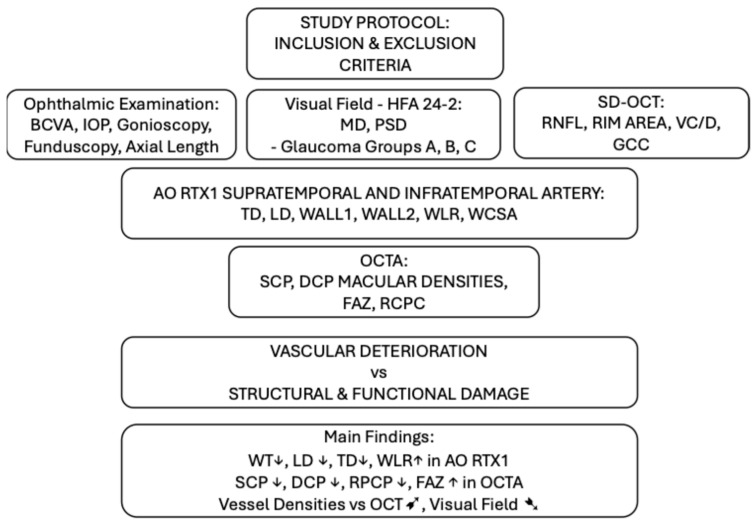
Data processing pipeline for examining each parameter in the retinal microcirculation study in glaucoma. BCVA—best-corrected visual acuity; IOP—intraocular pressure; MD—mean deviation in perimetry; PSD—pattern standard deviation in perimetry; SD-OCT—spectral domain optical coherence tomography; RNFL—retinal nerve fibre layer in SD_OCT; VC/D— vertical cup to disc ratio in OCT; GCC—ganglion cell complex; AO-RTX1—Adaptive Optics Fundus Camera RTX1; TD—total diameter in AO-RTX1; LD—lumen diameter in AO-RTX1; WALL1—first wall thickness in AO-RTX1; WALL2—second wall thickness in AO-RTX1; WLR—wall to lumen ratio in AO-RTX1; WCSA—wall cross-sectional area in AO-RTX1; OCTA—optical coherence tomography angiography; SCP—superficial plexus capillary density; DCP—deep plexus capillary density; ↑—parameter value increase; ↓—parameter value decrease; ➹—positive correlation;➷—negative correlation.

The present study aimed to evaluate the parameters of retinal arterioles using rtx1™ AO-FC and peripapillary and macular vessel densities with OCTA in eyes with primary open-angle glaucoma (POAG) at different stages compared to healthy eyes. Secondly, the associations between these vascular parameters and glaucoma severity were investigated, as defined by structural changes in OCT and functional changes in the VF test, along with comparisons with results in healthy eyes. This study is the first trial to analyse retinal microcirculation with two modern non-invasive methods in correlation with structural and functional tests in POAG. The data processing pipeline for examining each parameter is illustrated in Figure 1, which outlines the parameters assessed in the study of retinal microcirculation in glaucoma.

## 2. Materials and Methods

This study was performed between July and September 2022 at the Department of Ophthalmology, Faculty of Medicine, the Medical University of Warsaw, in the Ophthalmic Public Hospital in Warsaw. The Bioethical Commission for the Medical University of Warsaw approved the study protocol (approval number KB/87/2015, 7 April 2025). All investigations were conducted in accordance with the principles outlined in the Declaration of Helsinki. After the presentation of the study protocol, all participants signed an informed consent form. The study was designed as a cross-sectional, single-centre study.

### 2.1. Inclusion Criteria

The patients included in the study had been diagnosed with bilateral POAG, with an assessment of retinal nerve fibre layer (RNFL), ganglion cell complex (GCC) defects, and ONH parameters using optical coherence tomography (OCT), mean deviation (MD), and pattern standard deviation (PSD) in perimetry. Glaucoma was diagnosed at least two years ago.

Glaucoma in each eye was staged, depending on the results of the last reliable perimetry test, using the simplified criteria of Hodapp’s classification [3] as follows:

1. Glaucoma A–pre-perimetric glaucoma: no visual field scotoma in perimetry, MD greater than 6 dB (16 eyes);
2. Glaucoma B–early perimetric glaucoma, MD less than −6 dB (24 eyes);
3. Glaucoma C–moderate perimetric glaucoma, MD less than −12 dB (17 eyes).

### 2.2. Exclusion Criteria

- refractive errors > 6 D or cylindrical lens ≥ 2.5 D,
- axial eye length ≥ 26 mm,
- distance best-corrected visual acuity (BCVA) ≤ 0.4 on Snellen charts,
- media opacities resulting in low image quality,
- diabetes mellitus,
- history of trauma,
- any other ocular diseases that may influence retinal vessel morphology.

### 2.3. Study Protocol

Demographic data, including age, weight, height, and a history of general diseases (hypertension, diabetes, ischemic heart disease, or stroke), were obtained from questionnaires completed by the participants. We checked the systolic and diastolic blood pressures (SBP, DBP) in the sitting position on the brachial artery after a 5 min rest.

The full ophthalmic examination, including distance best-corrected visual acuity (BCVA), refractometry, slit lamp biomicroscopy, Goldmann applanation tonometry, gonioscopy, and direct fundoscopy was performed. Axial eye length was acquired using the IOL Master 700 (Carl Zeiss Meditec AG, Hennigsdorf, Germany). The RNFL, GCC thickness, and rim area were assessed using spectral domain optical coherence tomography (SD-OCT) with the RTVue XR 100 Avanti Edition (Optovue Inc., Fremont, CA, USA).

Humphrey 24.2 sita-standard visual field with the reliability indices of the European Glaucoma Society was performed on Humphrey Field Analyzer 3 (Zeiss, Germany).

AO fundus images were obtained using AO-FC (Rtx1™; Imagine Eyes, Orsay, France) with AO Detect Artery software (version 3.4) to analyse retinal vessel parameters [6]. Examinations were conducted without pupil dilation. Images of a superior and inferior temporal retinal artery were obtained. The following parameters were assessed: lumen diameter (LD), wall thickness (WT), and total diameter (TD). The following two parameters, wall-to-lumen ratio (WLR) and cross-sectional area of the vascular wall (WCSA), were automatically obtained from the AO artery detection software. WLR is the ratio of the vessel’s WT to LD, calculated as 2 × WT ÷ LD, while WCSA describes the relationship between LD and TD. All the above-mentioned retinal parameters were measured three times on the scan with the best quality; the arithmetic mean of these three values was used in the statistical analysis.

Angio-OCT (OCTA) images were obtained using spectral optical tomography (SD-OCT) (REVO NX, software version 11.07, Optopol Technology, Zawiercie, Poland) with an angio-OCT option. The REVO NX is characterised by a light source of 850 nm wavelength and 50 nm bandwidth (half-bandwidth) utilising SLED technology. It operates at a scanning speed of 130,000 measurements per second, achieving a digital axial resolution of 2.8 μm and 5.0 μm within the tissue. The transverse resolution is 12 μm, with a typical value of 18 μm. Macular vasculature was evaluated on 6 × 6 mm scans of the Superficial and Deep Plexus Capillary (SCP, DCP). The measurement zones included the following regions: total (entire measurement area), superior (upper half of the measurement area), inferior (lower half of the measurement area), centre (central ring with a diameter of 1 mm), and the combined area (ETDRS) of three concentric rings with diameters of 1, 3, and 6 mm. Additionally, the foveal avascular zone (FAZ) was evaluated within the Superficial and Deep Plexus of the macula in semi-automatic mode.

The following parameters were assessed: FAZ area in mm^2^, perimeter of the FAZ area in mm, and circularity of the FAZ area (the ratio of the measured perimeter to the circumference of a circular area of the same size). Angio-OCT images of 4 × 4 mm were used to assess ONH vascularisation. Quantitative measurements (the density map) were performed within the radial peripapillary capillary plexus (RPCP) in total (entire measurement area), superior (upper half of the measurement area), and inferior (lower half of the measurement area) regions. REVO’s real-time hardware eye-tracking feature compensated for blinking, loss of focus, and unintentional eye movements during OCT scanning [26]. Only a few patients required manual scanning after multiple attempts in automatic mode. Dilating the pupils was unnecessary to obtain an optimal-quality image.

### 2.4. Statistical Analyses

The data analysis was conducted with Statistica™ v. 13.2, TIBCO Software Inc., Palo Alto, CA, USA; 2017. For continuous variables, the presented means with their standard deviations (SD) were compared between the POAG and control groups using either Student’s *t*-test or the Mann–Whitney *U*-test, depending on the data distribution. The Shapiro–Wilk test was used to determine the normality of each continuous variable. Kruskal–Wallis and chi-square tests were used to compare at least three glaucoma groups on a quantitative variable. Relationships between numerical variables were assessed using Pearson correlation analysis when the data met parametric conditions for a parametric test and Spearman correlation analysis when they did not. A two-sided test was applied for *p*-values, and statistical significance was defined as a *p*-value of less than 0.05.

## 3. Results

Subjects: We included 31 patients with 62 eyes with primary open-angle glaucoma (POAG), recruited from the Glaucoma Department at the Ophthalmic Public Hospital in Warsaw, and 25 healthy volunteers with 50 eyes. Five eyes from the study group did not meet the inclusion criteria: three were excluded due to excessive axial length, one had a history of ocular trauma with distance visual acuity less than 0.4 on Snellen charts, and one had undergone a complicated glaucoma surgery.

Prior and ongoing glaucoma treatment:

Seventeen eyes had undergone previous glaucoma surgeries alone (eight eyes) or in combination with phacoemulsification (nine eyes), and eight eyes had undergone selective laser trabeculoplasty (SLT) at least 1 year prior to the study. Most patients were on politherapy with two or more antiglaucoma agents (23 eyes), while 17 eyes were treated as monotherapy, and the same number of eyes received no antiglaucoma drops.

Patients from the control group had an IOP of less than 21 mmHg and a normal appearance of the ONH, RNFL, and GCC parameters, all of which were within normal ranges, as determined by OCT.

All subjects were above 18 years old and were of white European descent.

### 3.1. Glaucoma vs. Healthy Eyes

Significant differences existed between the control (healthy) and glaucoma groups in terms of best-corrected visual acuity (BCVA) and intraocular pressure (IOP). There were no differences between groups in age (*p* = 0.067), systolic and diastolic blood pressures (SBP, *p* = 0.117; DBP, *p* = 0.069), and BMI (*p* = 0.069). Patients with glaucoma had lower distance BCVA (0.998 vs. 0.895 for the healthy and glaucomatous groups, respectively, *p* < 0.001) and lower IOP values (15.08 vs. 13.2 in the healthy and glaucomatous groups, respectively, *p* < 0.001). There were similar percentages of patients suffering from arterial hypertension (HA) (*p* = 0.896) or coronary heart disease (CHD) (*p* = 0.346) between study groups. Mean diastolic systemic blood pressure (DBP) was not significantly lower in the glaucoma group (80.5 vs. 77.7 for healthy and glaucomatous groups, respectively; *p* = 0.069). Significant differences, confirming the group classification, were found in all analysed OCT parameters: mean RNFL, mean GCC, and VC/D ratio (*p* < 0.001). The results are presented in Table 2.

The glaucoma groups were compared regarding OCT parameters (mean RNFL, vertical c/d ratio, mean GCC, and rim area), visual field parameters (mean deviation (MD) and pattern standard deviation (PSD)), retinal artery parameters in AO rtx1™, and OCTA measurements.

Table 3 evaluates the OCT parameters, including rim area, RNFL, GCC, and vc/d, in three glaucoma groups. There were significant differences among all glaucoma subgroups, depending on the severity of the disease. The RNFL thickness, as well as the average thickness in the superior and inferior quadrants, rim area, and average GCC thickness, progressively decreased with the advancement of optic nerve damage. The values of the vertical c/d ratio increased.

The perimetry test results also changed with the progression of glaucoma, starting from MD and PSD values within normal limits in group Glaucoma A to their values in the Glaucoma C group, which classified the eyes as moderately damaged in the Hodapp classification. The results are presented in Table 4.

### 3.2. Comparison of AO Rtx1 Arteriolar Parameters Between Glaucoma and Healthy Eyes

All analysed arteriole parameters, except the mean WCSA of the inferior artery, showed significant differences between healthy and glaucoma eyes. The wall thicknesses (WT1, WT2) were significantly higher in eyes with glaucoma than in the control group. Since the mean lumen diameter (LD) values were significantly smaller in all glaucoma groups than in the control group, it turned out that there were increased values of the WLR ratio in the glaucoma group for the supratemporal artery (0.301 vs. 0.238 for glaucomatous and healthy groups, respectively) and for the infratemporal artery (0.308 vs. 0.241 glaucomatous for and healthy groups, respectively (*p* < 0.001)). The mean total diameter (TD) values were significantly smaller in glaucoma eyes compared to healthy eyes (*p* < 0.001) (Table 5).

### 3.3. Comparison of AO Rtx1 Arteriolar Parameters Between Glaucoma Groups

The following AO rtx1™ arteriolar parameters did not show statistically significant differences between glaucoma groups: vessel wall thickness, total diameter, and WCSA for the supratemporal (*p* > 0.05) and infratemporal (*p* > 0.05) retinal arterioles. Decreasing of the mean supratemporal artery lumen was observed with the progression of GON, but it was not found in the inferior localisation of the analysed artery. WLR did not differ significantly between glaucoma groups, but the mean values were slightly higher in groups Glaucoma B and Glaucoma C compared to Glaucoma A (Table 6).

### 3.4. Comparison of OCTA Parameters Between Glaucoma and Healthy Eyes

Both total, superior, and inferior Superficial Plexus Capillary (SCP) densities in the macula region were significantly lower in the glaucoma group compared to the control group. In comparison, significant differences in total and inferior Deep Plexus Capillary (DPC) macular densities were found between groups. The density in the superior DCP was lower in glaucomatous eyes; however, the difference was not statistically significant (*p* = 0.073). The combined area (ETDRS) of three concentric rings of 1, 3, and 6 mm diameters was significantly lower in the superficial plexus and not significantly different in the deep plexus in glaucoma eyes (*p* < 0.001 for SCP ETDRS; *p* = 0.09 for DCP ETDRS).

Additionally, the FAZ was evaluated within the superficial and deep plexus of the macula, and it was found to be significantly larger in eyes with glaucoma compared to those of healthy individuals. Other parameters assessed in the FAZ also showed significant differences between groups: the perimeter of the FAZ area was enlarged in the glaucoma group in SCP and DCP, and the circularity of the FAZ area was lower in the glaucoma group.

Quantitative measurements of the ONH’s vascularisation were performed within the radial peripapillary capillary plexus (RPCP). Compared to the control group, eyes with glaucoma had significantly lower peripapillary vessel densities in all analysed regions: total, superior, and inferior (*p* < 0.001) (Table 7).

### 3.5. Comparison of OCTA Parameters Between Glaucoma Groups

The SCP macular densities were higher in eyes with early glaucoma (group Glaucoma A) compared to groups Glaucoma B and Glaucoma C, which had mild and severe GON, respectively. Significant differences were observed in total and inferior SCP macular densities between glaucoma eyes (*p* = 0.014, *p* = 0.004, respectively). There were no differences in the DCP macular densities between glaucoma subgroups, regardless of the disease stages. The FAZ in the superficial plexus did not differ between groups Glaucoma A and Glaucoma C. Still, a non-significant difference was found in the deep plexus in the mentioned subgroups. The vascularisation around the ONH (RPCP) was the highest in eyes with early glaucoma (Glaucoma A) and decreased significantly in groups Glaucoma B and Glaucoma C in all analysed regions (Table 8).

### 3.6. Correlations of the OCTA Results with Other Analysed Parameters

A negative correlation was found between SCP values and systolic SBP and diastolic DBP blood pressures in healthy eyes, with respective correlation coefficients of *r* = −0.432 and *r* = −0.340. A similar relationship was not found in any of the subgroups of glaucomatous eyes. In the control group, no other correlations were found between the SCP and DCP values, OCT parameters (RNFL, rim area, VC/D, and GCC), and arteriole parameters in AO rtx1™.

In glaucoma eyes, SCP macular density was positively correlated with OCT parameters (RNFL, *r* = 0.621; GCC, *r* = 0.536; rim area, *r* = 0.489) and VF parameters (MD, *r* = 0.426), and negatively correlated with PSD and WLR from AO rtx1™ (*r* = −0.517). This suggests that better structural and functional integrity is associated with higher SCP density. In the whole glaucoma group, there were positive correlations between SCP macular density and vessel size (TD) (*r* = 0.471), vessel lumen (LD) (*r* = 0.494), and vessel surface area (WCSA) (*r* = 0.342), and a negative correlation with WLR (*r* = −0.476) in AO rtx1™.

A positive and significant correlation was observed between SCP macular density and RNFL in the glaucoma subgroups A and B (*r* = 0.680 in both). However, it did not occur in the C group, which consisted of eyes with more advanced glaucoma. Glaucoma subgroups B and C also demonstrated a negative correlation of SCP macular density with WLR (*r* = −0.530 and *r* = −0.518, respectively) in AO rtx1™. Subgroup Glaucoma C showed a positive correlation between SCP and LD in AO rtx1™ (*r* = 0.521).

The correlation analysis of DCP macular density in glaucomatous eyes revealed a positive relationship with OCT parameters, specifically RNFL (*r* = 0.387), rim area (*r* = 0.335), and GCC (*r* = 0.390). Still, no correlation was found between the parameters of the visual field and the retinal arterioles in AO rtx1™.

#### Assessment of the correlation of the RPCP

The density of the RPCP in healthy and glaucomatous eyes was positively correlated with diastolic blood pressure (*r* = 0.432). RPCP density in glaucoma eyes correlated positively with RNFL (r = 0.334) and GCC thickness (*r* = 0.457) in OCT, and MD (*r* = 0.480) in VF, and negatively with PSD (*r* = −0.375) in VF and arterial wall thickness (*r* = −0.489) from AO rtx1™. The analysis of individual subgroups of glaucomatous eyes confirmed a negative correlation between the thickness of the retinal artery walls (WT) in AO rtx1™ and RPCP in Glaucoma B (*r* = −0.567). Positive correlations were found with OCT and MD in VF for eyes from Glaucoma C (*r* = 0.722).

## 4. Discussion

Glaucoma is a chronic neuropathy in which RGCs die, causing irreversible defects in the visual field [2]. According to the vascular theory of glaucoma development, retinal microcirculation disorders play a very important role in both the pathogenesis and progression of GON [5,6,7]. Impaired blood flow in the optic nerve ONH and retina in glaucoma patients is well documented [7,8,21,22,23,24,25,27,28,29,30,31].

Our study, published in 2024 in *JCM* [6], is significant as it revealed changes in retinal vessels, using adaptive optics, in eyes with very early glaucoma, a stage previously difficult to detect without visual field defects. This finding, unique to our study, underscores the importance of analysing the relationship between retinal microcirculation disorders and the severity of functional and structural changes during glaucoma. Some studies have investigated the relationship between retinal microcirculation disorders and the severity of glaucoma [23,24,25]. However, our study is the first to evaluate two modern non-invasive imaging methods, OCTA and AO rtx1™, for retinal microcirculation analysis.

Our study employed a comprehensive approach, comparing patients with different stages of glaucoma (GON) with a control group of healthy eyes and within the glaucoma subgroups, depending on the severity of the disease. We also performed a correlation analysis of the changes in retinal microcirculation with parameters illustrating structural (OCT) and functional (VF) damage in glaucoma.

OCTA and AO are powerful complementary techniques that have fundamentally changed retinal and optic nerve head (ONH) imaging. They enable the routine visualisation of structures with cellular resolution in either cross-sectional or en face views. OCTA allows visualisation of the density of superficial and deep vascular plexuses. At the same time, AO enables the determination of all structural parameters of retinal small arterioles, as it can distinguish the vessel walls and lumen from the blood column. This method distinguishes between the functional vasoconstriction of an arteriole and the structural remodelling of a vessel [15].

### 4.1. AO rtx1™

Significant differences were found in all analysed retinal arterioles’ parameters in AO between glaucoma and healthy eyes. AO imaging revealed early structural changes in retinal arterioles, such as narrowing, wall thickening, and increased WLR, even in pre-perimetric glaucoma. Previously, the narrowing of retinal arterioles in POAG has been demonstrated in several studies [24,31,32,33,34,35]. Our study’s results are similar to those of Hugo et al., who was the first to use AO to evaluate the retinal vasculature in glaucoma and found a significant reduction in TD and LD in POAG patients compared to healthy individuals [36]. While vessel wall thickness and total diameter did not differ significantly between glaucoma stages, a trend of decreasing artery lumen was observed with GON progression.

WLR was significantly higher in all examined eyes with glaucoma; its values increased slightly with the progression of GON. The reduction in vessel calibre and lumen in more severe glaucoma ONH damage was not accompanied by an increase in the thickness of the arterial walls. It may indicate a lack of hypertrophy, a characteristic of hypertensive retinopathy [37]. Signs typical for eutrophic remodelling, characterised by an increased WLR and reduced LD due to arterioles vasoconstriction [37], were found in our study. This was also confirmed by the lower average WCSA in the entire group of eyes with glaucoma compared to the control group. This parameter indicates the vessel’s surface area and increases in vascular diseases associated with thickening and hypertrophy of the blood vessel walls, e.g., arterial hypertension or diabetes [37].

### 4.2. OCTA: SCP, DCP, RPCP, FAZ

OCTA demonstrated reductions in microvascular density in the macular and peripapillary regions, correlating with the severity of glaucoma and functional loss. Our results are consistent with other studies that analysed retinal vessel density using angio-OCT in glaucomatous eyes [21,22,23,24,25,29,30]. Studies using non-invasive techniques, such as Laser Doppler flowmetry and laser speckle flowgraphy, also confirmed lower blood flow on the entire optic disc in glaucoma eyes [27,28]. The SCP macular densities were higher in eyes with early glaucoma (group A) compared to eyes with mild and severe GON. There were no differences in the DCP macular densities between glaucoma subgroups, regardless of the disease stages. The vascularisation around the ONH (RPCP) was highest in eyes with early glaucoma and decreased significantly in groups B and C in all analysed regions. Enlarged FAZ areas indicate ischemic microvascular changes in glaucoma, and we observed a correlation between these areas and disease severity. FAZ area has been implicated in pathological processes like diabetic retinopathy [38,39]. Enlarged FAZ areas have also been described in glaucoma eyes, exhibiting a similar diagnostic value for glaucoma as conventional structural parameters analysed in OCT (RNFL and GCC thickness) [38,40,41,42].

The enlarged FAZ area in glaucoma eyes ranged from 0.360 to 0.435 mm^2^, depending on the type of OCTA machine, segmentation method, and image analysis approach [40,41,42]. In our study, which examined only Caucasian patients, the mean FAZ area was 0.295 and 0.546 mm^2^ for healthy and glaucoma eyes, respectively—a value even slightly higher than that reported in earlier studies for the Asian population. Kwon et al. reported an enlarged FAZ area, which correlated with VF defects in patients with glaucoma [41,42]. They presented results consistent with ours, showing that an enlarged FAZ area was also found in glaucomatous eyes without visual field defects [40]. Shoji et al. found that IOP reduction after glaucoma surgery improved perifoveal microcirculation and decreased FAZ area up to 3 months postoperatively [38]. Their results support the hypothesis of RGC-function reversibility with IOP reduction [38]. They concluded that OCTA-derived FAZ metrics may be biomarkers for RGC activity and visual function [38].

### 4.3. SCP Correlations with OCT, VF, AO rtx1™

There were positive correlations in glaucoma eyes between SCP macular density and OCT parameters (RNFL, GCC, rim area) and VF (MD), and negative correlations with PSD. Thicker RNFL, GCC, and larger rim areas were found in eyes with higher SCP density. In the analysis of individual subgroups of eyes with glaucoma, a positive correlation was observed between SCP density and RNFL in OCT only in eyes with early and moderate glaucoma, but not in eyes with advanced glaucoma. This may indicate a lower demand for nutrients and capillary vascularisation in retinal areas with RGC damage. Similar to our results, Rao et al. demonstrated that macular vessel density in the parafoveal region was reduced as measured in patients with glaucoma compared to healthy eyes [43]. Shoji et al. suggested that changes in macular vessel density may be used for monitoring GON progression [44]. On the other hand, Rao et al. postulated that macular vessel density has moderate diagnostic performance, which is poorer than that of measurements in the peripapillary region, in differentiating glaucoma eyes from healthy eyes. Their study included individuals with pre-perimetric and early glaucoma whose foveal region remains intact, which could be responsible for the poorer diagnostic accuracy of macular vessel density measurements [43].

Yarmohammadi et al. demonstrated that vessel densities in both the peripapillary and macular regions were significantly lower in the eyes of patients with POAG and unilateral visual field loss compared to healthy eyes of a similar age [45]. The peripapillary vessel density in the eyes with VF defect was lower than in their fellow unaffected eyes. In contrast, the difference in macular vessel density was similar in both eyes of the same patient [45]. In another study published in 2017, the same authors reported that deterioration of peripapillary and macular vessel density was also found in perimetrically intact glaucomatous eyes [46]. This finding is consistent with our results, which show significantly lower vessel density measurements even in eyes without detectable visual field damage in early glaucoma. Another similarity in our study concerned OCT parameters, including RNFL, rim area, and GCC, which were significantly lower even in glaucomatous eyes without visual field defects than in healthy eyes [45]. Larger SCP macular densities corresponded with larger arteriole lumen and total diameters and lower WLR in AO rtx1™. DCP densities correlated only with OCT parameters, not AO-measured arteriole parameters.

### 4.4. RPCP Correlations with OCT, VF, AO rtx1™

RPCP density positively correlated with RNFL, GCC in OCT, and VF MD, and negatively correlated with VF PSD and arteriole wall thickness in AO rtx1™.

A correlation between reduced RPCP density and structural and functional glaucoma defects was also found in other studies [21,22,23,24,25]. Wang et al. reported that decreased disc flow index and peripapillary vessel density in glaucomatous eyes correlated with the severity of glaucoma damage [25]. The RNFL primarily comprises the axons of RGCs and has high metabolic requirements that depend on regional capillary networks running parallel to the RGC axons [22]. Akagi et al. suggested that peripapillary microvascular reduction may occur after retinal nerve fibre layer (RNFL) thinning [23]. Still, other authors found lower retinal capillary perfusion even before the apoptotic death of defective retinal ganglion cells [24,25]. Yarmohammadi et al. found that capillary vessel density has comparable diagnostic accuracy to RNFL thickness in OCT scans for detecting glaucoma and glaucoma suspects [29]. They did not classify glaucoma eyes for GON advancement as we did. The mean VF parameters (MD: −3.9 dB (−8.8, 1.8)) and PSD: 4.6 dB (2.7, 8.9)) and average RNFL thickness (74.5 (72.1–76.9)) allow patients to be classified as having early and mild GON [29]. In our study, we divided the eyes of glaucoma patients into three subgroups, ranging from early pre-perimetric glaucoma to those with severe defects in OCT and visual field tests. Quantitative measurements of the vascularisation of the ONH showed significantly lower values in all analysed regions in all glaucoma subgroups.

The VF MD correlates more strongly with peripapillary vessel density than with RNFL thickness or rim area, suggesting that vascular perfusion may correlate more closely with visual function [24]. In the study conducted by Yospon, the correlation between VF parameters and RPCP was moderate to good [47]. Yospon et al. also reported significantly lower peripapillary vessel densities in eyes with severe glaucoma than in those with early and moderate glaucoma. Their values were strongly correlated with average RNFL thickness but only moderately related to the GCC, as these parameters are measured in different anatomical areas [47]. Our study showed no significant differences between superior and inferior RPCP localisations and decreased vessel densities in the group with early glaucoma (Glaucoma A) compared to the subsequent subgroups, but without differences between Glaucoma B and Glaucoma C.

In the correlation analysis of two retinal vessel imaging methods, AO rtx1™ and OCTA, higher SCP densities were correlated with wider arterial vessels (TD), larger vessel lumens (LD), and lower WLR coefficients. In eyes with reduced SCP density, higher WLR values were found. In the eyes of the group Glaucoma C, reduced SCP density was also correlated with the narrowing of the arterial lumen. These findings suggest a strong relation between the superficial capillary plexus and the morphology of small retinal arterioles.

When considering future directions for OCTA and AO-based technologies, it is impossible to overlook the growing role of artificial intelligence (AI), whose applications span from early detection to disease progression assessment and outcome prediction. AI has demonstrated its utility across various ophthalmic modalities, including perimetry, fundus photography, and OCT [48,49]. AI has also been explored in OCTA, where it has shown high diagnostic performance, even in complex cases such as highly myopic glaucoma [50,51]. In the study by Lee et al., a deep learning (DL) model was developed using macular OCTA images, achieving an area under the curve (AUC) of 0.946, which is comparable to that of macular OCT-based models [50]. Moreover, other studies demonstrated that OCTA-based machine learning (ML) algorithms can perform similarly or even better than conventional OCT-derived parameters in classifying glaucoma severity [51]. AI integration with adaptive optics OCT (AO-OCT) has also been reported, where DL algorithms were successfully applied to identify and segment RGCs at the cellular level, offering unprecedented high-resolution insights into glaucomatous damage [52]. In the future, the integration of AI with advanced imaging techniques is expected to significantly enhance the accuracy, sensitivity, and clinical utility of glaucoma diagnostics and monitoring [53]. However, further studies are needed to validate these approaches.

## 5. Limitations

Due to the study’s non-interventional design, it was not possible to evaluate the potentially confounding impact of ocular hypotensive eye drops and blood pressure-lowering medications on vascular measurements. It is also worth noting that, in the present study, patients were not excluded based on systemic conditions, except diabetes, or medications, to better reflect the general population. Further studies and larger study groups are needed to investigate the influence of ocular hypotensive treatment and systemic medications on OCTA and AO rtx1™ measurements. Larger, longitudinal studies are required to clarify and validate these imaging biomarkers.

Since glaucoma prevalence and response to treatment can vary among ethnicities, including a more diverse sample population would enhance the applicability of the findings, as well as the generalisability and significance of future multicentre studies.

Another limitation of this study is the technical limitations of the imaging devices used. Among the known limitations of OCTA are projection artefacts resulting from superficial retinal vessels casting flow signals onto deeper layers and segmentation inaccuracies, particularly in the presence of retinal pathologies such as intraretinal fluid, pigment epithelial detachments, or atrophy. Moreover, eye movements during image acquisition can introduce motion artefacts. Another limitation is the inability of OCTA to visualise vascular leakage or dynamic perfusion phenomena. Furthermore, significant variability exists between different OCTA devices due to differences in hardware, limiting cross-platform comparability. Quantitative OCTA metrics, such as vessel density or flow void area, are also sensitive to factors like slab definition, signal strength, and projection methods, which may affect their reproducibility and clinical validity. Although our study focused on central retinal structures, where OCTA is particularly well suited, its inherently limited field of view restricts the evaluation of peripheral retinal vasculature [20]. Adaptive optics (AO) imaging also presents several limitations. These include high operational costs and the absence of normative databases necessary for clinical interpretation. AO is highly sensitive to media opacities such as cataracts, corneal scarring, or vitreous debris, as well as to patient cooperation and eye movements, all of which can significantly compromise image quality. Moreover, AO systems typically provide a very narrow field of view, limiting the ability to perform consistent longitudinal imaging of the same retinal area or to assess peripheral retinal pathology. Importantly, AO delivers only structural information, without direct correlation to functional status. In addition, the axial resolution of AO fundus cameras remains inferior compared to that of AO-OCT and scanning laser ophthalmoscopy (SLO), which may limit its utility in resolving fine depth-related retinal features [15].

## 6. Conclusions

This study confirms that vascular alterations in retinal arterioles and capillary plexuses are associated with the pathophysiology and progression of glaucoma. Our results support the concept of vascular deterioration in glaucoma, where vascular changes may precede structural abnormalities in the optic nerve head (ONH). Reduced retinal vessel densities in the macular and peripapillary regions, as well as narrowing of retinal arterioles, correlate with structural loss and visual field defects in glaucomatous eyes. Enlarged FAZ areas indicate ischemic microvascular changes in glaucoma. These data suggest that both OCTA and AO rtx1™ measurements show a very good correlation with the severity of glaucoma, allowing for the discrimination of eyes with glaucoma from healthy eyes earlier than in typical diagnostic glaucoma tests. They may be useful tools in the early diagnosis, staging, and monitoring of patients with glaucoma, as well as in identifying eyes at high risk before irreversible damage occurs. The combined use of AO rtx1™ and OCTA provides complementary and detailed insights into retinal microcirculation, surpassing the limitations of traditional imaging.

## Figures and Tables

**Table 1:** Diagnostic non-invasive technologies for assessing retinal vasculature in glaucoma.

Technology	Evaluated Parameters
Scanning Laser Doppler Flowmetry (SLDF) (Heidelberg Retina Flowmeter; Heidelberg Engineering, Heidelberg, Germany)	Volumetric total capillary blood flow and flow velocity in the retina, wall-to-lumen ratio (WLR), wall cross-sectional area (WCSA), wall thickness (WT), lumen diameter (LD), total diameter (TD) [11,12,13]
Retinal Vessel Analyzer (RVA) (Dynamic Vessel Analyzer, Imedos Systems UG, Jena, Germany)	Dynamic vessel diameter response to flickering light, arterial and venous calibres [14]
Adaptive Optics Fundus Camera (AO-FC) (Rtx1, Imagine Eyes, Orsay, France)	Wall-to-lumen ratio (WLR), wall cross-sectional area (WCSA), wall thickness (WT), lumen diameter (LD), and total diameter (TD) [15,16]
Retinal Oximeter (RO) (Oxymap T1, Oxymap ehf., Reykjavik, Iceland)	Oxygen saturation of haemoglobin in retinal arterioles and venules [17]
Laser Doppler Velocimeter (LDV) (CLBF 100, Canon Inc., Tokyo, Japan)	Peak blood flow velocity in major retinal vessels [14]
Optical Coherence Tomography Angiography (OCTA) (Revo NX 130, Optopol Technology Sp. z o.o., Zawiercie, Poland); (AngioVue Imaging System, Optovue Inc., Fremont, CA, USA); Cirrus HD-OCT with AngioPlex, Carl Zeiss Meditec AG, Jena, Germany); (DRI OCT Triton, Topcon Corp., Tokyo, Japan).	Vessel density (VD), perfusion density, foveal avascular zone (FAZ), radial peripapillary capillaries (RPC), macular capillary plexus density [18,19,20,21,22]

**Table 2:** Demographic and OCT parameters in healthy (control group) and glaucoma groups.

Parameters	Control Group	Glaucoma Group	*p*-Value *
age (m ± SD) (years)	49.5 ± 4.4	51.7 ± 6.4	0.067
HA	8.3%	9.8%	0.896
CHD	4.2%	14.8%	0.346
BMI (m ± SD) (kg/m^2^)	23.7 ± 2.1	24.4 ± 1.8	0.069
SBP (m ± SD) (mm Hg)	123.4 ± 11.2	127.5 ± 10.1	0.117
DBP (m ± SD) (mm Hg)	80.5 ± 9.2	77.7 ± 7.1	0.069
BCVA (m ± SD)	0.998 ± 0.014	0.895 ± 0.142	<0.001
IOP (m ± SD) (mm Hg)	15.1 ± 2.5	13.2 ± 2.7	<0.001 ^†^
AL (m ± SD) (mm)	23.8 ± 0.8	24.0 ± 1.2	0.683
OCT c/d v (m ± SD)	0.501 ± 0.153	0.811 ± 0.118	<0.001
OCT RNFL (m ± SD) (µm)	101.3 ± 7.4	78.8 ± 13.9	<0.001
OCT GCC (m ± SD) (µm)	97.7 ± 4.8	80.74 ± 11.8	<0.001

* Mann–Whitney U test, ^†^ Student’s *t*-test; m—mean; SD—standard deviation; BMI—Body Mass Index; SBP—systolic blood pressure; DBP—diastolic blood pressure; BCVA—best-corrected visual acuity; IOP—intraocular pressure; AL—axial length; n—number, OCT c/d v—optical coherence tomography cup to disc ratio vertical; OCT RNFL—optical coherence tomography retinal nerve fibre layer; OCT GCC—optical coherence tomography ganglion cells complex.

**Table 3:** OCT parameters (rim area, RNFL, GCC, and vc/d) in 3 glaucoma groups.

OCT Parameters	Group Glaucoma A	Group Glaucoma B	Group Glaucoma C	*p*-Value *
Total number of eyes	16	24	17	0.702 ^‡^
RIM AREA (m ± SD)	0.854 ± 0.304	0.824 ± 0.324	0.492 ^†^ ± 0.146 (*p* < 0.001)	<0.001
Vertical Cup-to-disc ratio (m ± SD)	0.556 ± 0.051	0.596 ± 0.021	0.863 ^†^ ± 0.806 (*p* < 0.001)	<0.001
RNFL average (m ± SD) (µm)	88.6 ± 11.4	82.1 ± 10.7	65.3 ^†^ ± 10.7 (*p* < 0.001)	<0.001
RNFL superior (m ± SD) (µm)	88.7 ± 13.7	84.1 ± 14.0	68.3 ^†^ ± 13.5 (*p* = 0.004)	<0.001
RNFL inferior (m ± SD) (µm)	89.1 ± 11.2	80.1 ± 10.1	62.1 ^†^ ± 9.7 (*p* < 0.001)	<0.001
GCC (m ± SD) (µm)	90.0 ± 8.8	83.0 ± 9.4	68.8 ^†^ ± 8.2 (*p* < 0.001)	<0.001

* Kruskal–Wallis rank sum test; ^†^ significant value vs. groups Glaucoma A and Glaucoma B; ^‡^ Fisher’s exact test; m—mean; SD—standard deviation; Glaucoma A—pre-perimetric glaucoma: no visual field scotoma in perimetry, MD greater than −6 dB; Glaucoma B—early perimetric glaucoma, MD less than −6 dB; Glaucoma C—moderate perimetric glaucoma, MD less than −12 dB, RNFL—retinal nerve fibre layer; GCC—ganglion cell complex.

**Table 4:** Perimetry tests parameters for MD and PSD in 3 glaucoma groups.

Visual Field Parameters	Group Glaucoma A	Group Glaucoma B	Group Glaucoma C	*p*-Value *
Total number of eyes	16	24	17	0.702 ^‡^
MD (m ± SD) (dB)	−0.17 ± 0.91	−1.89 ± 1.49	−13.55 ± 7.66 (*p* ^†^ < 0.001; *p* ^††^ = 0.014)	<0.001
PSD (m ± SD) (dB)	1.56 ± 0.25	3.55 ± 2.04	10.30± 3.75 (*p* ^†^ < 0.001)	<0.001

* Kruskal−Wallis rank sum test; ^†^ all combinations for groups Glaucoma A, Glaucoma B, and Glaucoma C; ^††^ significant value vs. groups Glaucoma A and Glaucoma B; ^‡^ Fisher’s exact test; m—mean; SD—standard deviation; Glaucoma A—pre-perimetric glaucoma: no visual field scotoma in perimetry, MD greater than −6 dB; Glaucoma B—early perimetric glaucoma, MD less than −6 dB; Glaucoma C—moderate perimetric glaucoma, MD less than −12 dB.

**Table 5:** AO rtx1™ parameters in control (healthy) and glaucoma groups.

AO rtx1™ Parameters	Control Group	Glaucoma Group	*p*-Value *
TDm S (m ± SD) (µm)	124.1 ± 12.4	111.0 ± 10.4	<0.001 ^†^
LDm S (m ± SD) (µm)	100.6 ± 11.1	85.5 ± 10.4	<0.001 ^†^
1 WTm S (m ± SD) (µm)	12.2 ± 1.3	12.8 ± 1.4	0.030
2 WTm S (m ± SD) (µm)	11.6 ± 1.2	12.6 ± 1.2	<0.001 ^†^
WLRm S (m ± SD)	0.238 ± 0.002	0.301 ± 0.04	<0.001
WCSAm S (m ± SD) (µm^2^)	4197.3 ± 657.9	3909.5 ± 531.6	0.019 ^†^
TDm I (m ± SD) (µm)	124.4 ± 12.0	114.4 ± 11.0	<0.001 ^†^
LDm I (m ± SD) (µm)	100.4 ± 10.9	87.7 ± 10.8	<0.001 ^†^
1 WTm I (m ± SD) (µm)	12.1 ± 1.3	13.4 ± 1.8	<0.001
2 WTm I (m ± SD) (µm)	11.9 ± 1.1	13.3 ± 1.74	<0.001
WLRm I (m ± SD)	0.241 ± 0.021	0.308 ± 0.047	<0.001
WCSAm I (m ± SD) (µm^2^)	4245.6 ± 690.9	4257.5 ± 812.1	0.937

* Mann–Whitney U test; ^†^ Student’s *t*−test; m—mean; SD—standard deviation; TDm S—mean total diameter of supratemporal artery; LDm S—mean lumen diameter of supratemporal artery; 1 WTm S—mean one wall thickness of supratemporal artery; 2 WTm S—mean second wall thickness of supratemporal artery; WLRm S—mean wall to lumen ratio of supratemporal artery; WCSAm S—mean wall cross-sectional area of supratemporal artery; TDm I—mean total diameter of infratemporal artery; LDm I—mean lumen diameter of infratemporal artery; 1 WTm I—mean one wall thickness of infratemporal artery; 2 WTm I—mean second wall thickness of infratemporal artery; WLRm I—mean wall to lumen ratio of infratemporal artery; WCSAm I—mean wall cross sectional area of infratemporal artery.

**Table 6:** AO rtx1™ parameters in 3 glaucoma groups.

AO rtx1™ Parameters	Group Glaucoma A	Group Glaucoma B	Group Glaucoma C	*p*-Value *
Total number of eyes	16	24	17	0.702 **^†^**
TDm S (m ± SD) (µm)	113.1 ± 11.8	111.6 ± 10.2	109.4 ± 8.7	0.384
LDm S (m ± SD) (µm)	87.8 ± 11.3	85.8 ± 10.2	84.0 ± 9.5	0.416
1 WTm S (m ± SD) (µm)	12.9 ± 1.7	12.9 ± 1.3	12.5 ± 1.1	0.225
2 WTm S (m ± SD) (µm)	12.5 ± 1.4	12.7 ± 1.2	12.5 ± 1.2	0.867
WLRm S (m ± SD)	0.293 ± 0.043	0.304 ± 0.048	0.301 ± 0.041	0.744
WCSAm S (m ± SD) (µm^2^)	3982.5 ± 657.3	3971.2 ± 500.3	3808.0 ± 394.2	0.539
TDm I (m ± SD) (µm)	113.2 ± 10.6	116.1 ± 12.2	112.7 ± 14.0	0.595
LDm I (m ± SD) (µm)	87.7 ± 9.5	89.3 ± 11.3	85.8 ± 12.2	0.558
1 WTm I (m ± SD) (µm)	13.0 ± 1.6	13.4 ± 2.2	13.6 ± 1.5	0.506
2 WTm I (m ± SD) (µm)	13.0 ± 1.8	13.5 ± 1.8	13.2 ± 1.6	0.503
WLRm I (m ± SD)	0.302 ± 0.042	0.306 ± 0.057	0.316 ± 0.040	0.656
WCSAm I (m ± SD) (µm^2^)	4114.3 ± 746.1	4349.1 ± 838.6	4218.9 ± 910.7	0.584

**^†^** Fisher’s exact test; * Kruskal−Wallis rank sum test; m—mean; SD—standard deviation; TDm S—mean total diameter of supratemporal artery; LDm S—mean lumen diameter of supratemporal artery; 1 WTm S—mean one wall thickness of supratemporal artery; 2 WTm S—mean second wall thickness of supratemporal artery; WLRm S—mean wall to lumen ratio of supratemporal artery; WCSAm S—mean wall cross-sectional area of supratemporal artery; TDm I—mean total diameter of infratemporal artery; LDm I—mean lumen diameter of infratemporal artery; 1 WTm I—mean one wall thickness of infratemporal artery; 2 WTm I—mean second wall thickness of infratemporal artery; WLRm I—mean wall to lumen ratio of infratemporal artery; WCSAm I—mean wall cross-sectional area of infratemporal artery; Glaucoma A—pre-perimetric glaucoma: no visual field scotoma in perimetry, MD greater than −6 dB; Glaucoma B—early perimetric glaucoma, MD less than −6 dB; Glaucoma C—moderate perimetric glaucoma, MD less than −12 dB.

**Table 7:** OCTA parameters in healthy (control) and glaucoma groups.

OCTA Parameters	Control Group	Glaucoma Group	*p*-Value *
SCP FAZ (m ± SD) (mm^2^)	0.295 ± 0.125	0.546 ± 0.299	<0.001
SCP perimeter (m ± SD) (mm)	2.006 ± 0.497	3.444 ± 1.494	<0.001
SCP circularity (m ± SD)	0.700 ± 0.082	0.555 ± 0.159	<0.001
DCP FAZ (m ± SD) (mm^2^)	0.430 ± 0.172	0.651 ± 0.303	<0.001
DCP perimeter (m ± SD) (mm)	2.394 ± 0.471	3.342 ± 0.896	<0.001
DCP circularity (m ± SD)	0.716 ± 0.072	0.591 ± 0.122	<0.001
SCP Total (m ± SD)	38.61 ± 1.31	36.78 ± 1.85	<0.001
SCP Super (m ± SD)	38.76 ± 1.17	36.91 ± 1.84	<0.001
SCP Infer (m ± SD)	38.40 ± 2.06	36.63 ± 2.63	<0.001
SCP Etdrs (m ± SD)	38.46 ± 1.41	36.39 ± 3.60	<0.001
DCP Total (m ± SD)	41.06 ± 1.21	40.17 ± 2.54	0.024
DCP Super (m ± SD)	41.07 ± 1.21	40.23 ± 2.60	0.073
DCP Infer (m ± SD)	41.05 ± 1.62	40.10 ± 2.75	0.025
DCP Etdrs (m ± SD)	41.33 ± 1.38	40.67 ± 2.51	0.090
RPCP Total (m ± SD)	39.27 ± 1.48	35.42 ± 4.97	<0.001
RPCP Super (m ± SD)	39.44 ± 1.17	35.59 ± 5.01	<0.001
RPCP Infer (m ± SD)	39.03 ± 2.24	35.17 ± 5.14	<0.001

* Mann–Whitney U test; m—mean; SD—standard deviation; SCP FAZ—superficial capillary plexus foveal avascular zone; SCP perimeter—superficial capillary plexus perimeter; SCP circularity—superficial capillary plexus circularity; DCP FAZ—deep capillary plexus foveal avascular zone; DCP perimeter—deep capillary plexus perimeter; DCP circularity—deep capillary plexus circularity; SCP Total—superficial capillary plexus total macular density; SCP Super—superficial capillary plexus superior macular density; SCP Infer—superficial capillary plexus inferior macular density; SCP Etdrs—superficial capillary plexus Etdrs macular density; DCP Total—deep capillary plexus total macular density; DCP Super—deep capillary plexus superior macular density; DCP Infer—deep capillary plexus inferior macular density; DCP Etdrs—deep capillary plexus Etdrs macular density; RPCP Total—radial peripapillary capillary plexus total optic nerve head density; RPCP Super—radial peripapillary capillary plexus superior optic nerve head density; RPCP Infer—radial peripapillary capillary plexus inferior optic nerve head density.

**Table 8:** OCTA parameters in 3 glaucoma groups.

OCTA Parameters	Group Glaucoma A	Group Glaucoma B	Group Glaucoma C	*p*-Value *
Total number of eyes	16	24	17	0.702 **^‡^**
SCP FAZ (m ± SD) (mm^2^)	0.564 ± 0.356	0.449 ± 0.190	0.566 ± 0.289	0.711
DCP FAZ (m ± SD) (mm^2^)	0.569 ± 0.219	0.588 ± 0.308	0.762 ± 0.344	0.904
SCP Total (m ± SD)	37.9 ± 1.2	36.8 ± 2.2	36.6 ± 0.9 *p* ^††^ = 0.011	0.014
SCP Super (m ± SD)	37.7 ± 1.4	36.9 ± 2.1	36.7 ± 1.4	0.171
SCP Infer (m ± SD)	38.2 ± 1.2	36.6 ± 3.1	36.5 ± 1.0 *p* ^††^ = 0.004	0.004
DCP Total (m ± SD)	40.7 ± 3.2	39.9 ± 3.0	40.5 ± 1.0	0.063
DCP Super (m ± SD)	40.6 ± 3.3	40.0 ± 3.1	40.3 ± 1.3	0.126
DCP Infer (m ± SD)	40.7 ± 3.2	39.8 ± 3.0	40.7 ± 1.1	0.157
RPCP Total (m ± SD)	37.7 ± 0.8	34.3 ± 6.6	34.7 ± 4.4 *p* ^††^ = 0.003	0.033
RPCP Super (m ± SD)	37.8 ± 1.2	34.2 ± 6.7	34.6 ± 4.6 *p* ^††^ = 0.013	0.017
RPCP Infer (m ± SD)	37.5 ± 1.5	34.2 ± 6.8	34.8 ± 4.5 *p* ^††^ = 0.035	0.041

* Kruskal−Wallis rank sum test; ^††^ significant value Glaucoma A group vs. Glaucoma C group; ^‡^ Fisher’s exact test; m—mean; SD—standard deviation; SCP FAZ—superficial capillary plexus foveal avascular zone; DCP FAZ—deep capillary plexus foveal avascular zone; SCP Total—superficial capillary plexus total macular density; SCP Super—superficial capillary plexus superior macular density; SCP Infer—superficial capillary plexus inferior macular density; DCP Total—deep capillary plexus total macular density; DCP Super—deep capillary plexus superior macular density; DCP Infer—deep capillary plexus inferior macular density; RPCP Total—radial peripapillary capillary plexus total optic nerve head density; RPCP Super—radial peripapillary capillary plexus superior optic nerve head density; RPCP Infer—radial peripapillary capillary plexus inferior optic nerve head density; Glaucoma A—pre-perimetric glaucoma: no visual field scotoma in perimetry, MD greater than −6 dB; Glaucoma B—early perimetric glaucoma, MD less than −6 dB; Glaucoma C—moderate perimetric glaucoma, MD less than −12 dB.

## Data Availability

The data are not publicly available due to privacy.
